# Supplementary material for: Tissue Bioconcentration Pattern and Biotransformation of Per-Fluorooctanoic Acid (PFOA) in Cyprinus carpio (European Carp)—An Extensive In Vivo Study
Source: Foods. 2023 Mar 27;12(7):1423. doi: 10.3390/foods12071423 (PMC10093588; doi:10.3390/foods12071423)
Supplement: Supplementary file 1 [file foods-12-01423-s001.zip › foods-2227952-supplementary.pdf]

foods

Tissue Bioconcentration Pattern and Biotransformation of Per-Fluorooctanoic Acid (PFOA) in *Cyprinus carpio* (European Carp)—An extensive in vivo study

Valentina Andreea Petre †, Florentina Laura Chiriac †, Irina Eugenia Lucaciu, Iuliana Paun, Florinela Pirvu, Vasile Ion Iancu, Laura Novac and Stefania Gheorghe *

National Research and Development Institute for Industrial Ecology—ECOIND, Drumul Podu Dambovitei 57-73, Sector 6, 060652 Bucharest, Romania
* Correspondence: stefania.gheorghe@incdecoind.ro
† These authors contributed equally to this work.

Table S1. Acute toxicity data.

Compound	Species	Aquatic organism	End point	Efect conc. (mg/L)	Toxicit y	References
PFOA	*Brachionus calyciflorus*	rotifer	NOEC	0.125	Chronic	[60]
	Chironomus plumosus	midge	NOEC	0.0098	Chronic	[61]
	Chironomus riparius	midge	LOEC	0.0098	Chronic	[62]
	Chydorus sphaericus	crustacean	EC_{50}	176	Acute	[63]
	Cipangopaludina cathayensis	mollusk	LC_{50}	740	Acute	[64]
	Cyclops sp.	crustacean	LOEC	70	Chronic	[65]
	Daphnia magna	crustacean	EC_{05}	180	Acute	[66]
	Daphnia pulicaria	crustacean	LC_{50}	204	Acute	[67]
	Dugesia japonica	worm	LOEC	15	Acute	[68]
	Lampsilis siliquoidea	mollusk	EC_{50}	165	Acute	[69]
	Ligumia recta	mollusk	EC_{50}	161	Acute	[69]
	Limnodrilus hoffmeisteri	worms	LC_{50}	568	Acute	[64]
	Macrobrachium nipponense	crustacean, shrimp	LC_{50}	367	Acute	[64]
	Moina macrocopa	crustacean	EC_{50}	199	Acute	[70]
	Neocaridina denticulata	crustacean, shrimp	LC_{50}	564	Acute	[71]
	Physella acutea	mollusk, snail	LC_{50}	672	Acute	[71]
	Cyprinus carpio	fish	LOEC	2	Chronic	[72]
	Pimephales promelas	fish	NOEC	74.1	Chronic	[73]
	zebrafish	fish	LC_{50}	499	Acutee	[74]

Table S2. The gradient elution program.

Time (min)	MeOH (%)	Flow rate (mL/min)	Gradient programe role
0.00	40	0.200	Analytical separation
1.50	40	0.200	
2.50	60	0.200	
12.50	80	0.200	
13.00	80	0.200	
13.10	40	0.200	Column reechilibration
23.00	40	0.200	

Table S3. MRM experimental conditions used for LC-MS/MS identification of PFOA and known metabolites in fish organs and tissue.

Analytes	tR (min)	MRM	Voltaj Fragmentor (V)	Colision energy (V)	Cell Accelerated Voltage (V)	Dwell time (msec)
PFBA	2.67	213→168.7 (Q)	60	4	0	250
PFPeA	6.14	263→219 (Q)	71	3	5	250
PFHxA	9.51	313→269 (Q)	80	3	6	200
		313→119 (q)	50	22	0	200
PFHpA	10.98	369.2→319 (Q)	65	5	4	200
		362.9→169 (q)	75	13	0	200
PFOA	12.51	412.9→368.9 (Q)	80	5	2	200
		412.9→169.0 (q)	70	14	0	200

Table S4. Recovery yields (R), matrix effect (EM) and limits of quantification (LOQ) obtained for PFOA and its metabolites in fish tissue.

Analytes	R(%)	EM (%)	LOQ(ng/g)
PFBA	71	29	0.08
PFPeA	82	41	0.02
PFHxA	92	91	0.06
FHpA	77	77	0.03
PFOA	82	63	0.08

Table S5. Initial characteristics of the specimens selected for the bioconcentration test (0 days).

Characteristics	Experiment 1 (10 μg/L)	Experiment 2 (100 μg/L)	Martor
Average weight (g)	35.10±9.67	43.2±8.15	39.82+8.19
Total batch weight (20 fish, g)	772.10	950.40	448.82
Average length without caudal peduncle (cm)	10.77±0.86	11.25±0.75	11.14±0.79
Average total length (including caudal peduncle) (cm)	13.65±1.18	14.53±0.90	14.15±0.81
Average body height (cm)	4.04±0.49	4.21±0.39	4.19±0.36

Table S6. Final characteristics of the specimens after exposure period (92 days).

Characteristics	Experiment 1 (10 µg/L)	Experiment 2 (100 µg/L)	Martor
Average weight	38±10.5	39.7±8.5	39.13±11.37
Average length without caudal peduncle	11.10±0.9	10.8±0.5	10.75±1
Average total length (including caudal peduncle)	14.39±1.2	14.3±0.6	14.19±1.07
Average body height	3.89±0.4	3.9±0.2	4.13±0.44

Table S7. PFOA concentration values determined in tissue and organs in Experiment 1 (10 μg/L PFOA).

Organs/test	Fish Scarification Campaigns							
	8w		12w		14w		17w	
	Conc (ng/g d.w.)	± SD	Conc (ng/g d.w.)	± SD	Conc (ng/g d.w.)	± SD	Conc (ng/g d.w.)	± SD
Gills	426	9.37	225	4.95	148	3.26	90.2	1.98
Scaly	286	6.18	342	7.39	370	7.99	3.12	0.07
Kidney	435	9.66	431	9.57	374	8.30	265	5.88
Muscle	57	1.43	375	9.41	370	9.29	0.69	0.02
Skin	318	7.54	345	8.18	353	8.37	4.87	0.12
Brain	270	6.59	275	6.71	190	4.64	58.1	1.42
Gonads	149	3.95	234	6.20	26.5	0.70	6.02	0.16
Liver	543	15.3	143	4.03	36.2	1.02	38.1	1.07
Intestine	97.8	2.57	246	6.47	83.3	2.18	46.4	1.21
Gallbladder	2572	65.3	1589	40.4	842	21.4	375	9.53

Table S8. PFOA concentration values determined in tissue and organs in Experiment 2 (100 μg/L PFOA).

Organs/test	Fish Scarification Campaigns							
	8w		12w		14w		17w	
	Conc (ng/g d.w.)	± SD	Conc (ng/g d.w.)	± SD	Conc (ng/g d.w.)	± SD	Conc (ng/g d.w.)	± SD
Gills	4222	92.9	2456	54.0	187	4.11	67.5	1.49
Scaly	1800	38.9	1261	27.2	87.1	1.88	15.5	0.33
Kidney	13581	301	2236	49.6	179	3.97	76.5	1.70
Muscle	981	24.6	1565	39.3	48.4	1.21	6.15	0.15
Skin	2526	59.9	760	18.0	100	2.37	23.1	0.55
Brain	1758	42.9	1118	27.3	78.5	1.92	12.8	0.31
Gonads	2443	64.7	1915	50.7	86.5	2.29	16.5	0.44
Liver	2729	77.0	745	21.0	112	3.16	42.4	1.20
Intestine	828	21.8	1136	29.9	95.3	2.51	51.1	1.34
Gallbladder	18640	473	8756	222	1026	26.1	446	11.3

Table S9. Concentration values (ng/g d.w.) of PFOA bio-transformation products determined in fish organs.

Sampling campagne	Experiment 1								Experiment 2							
	8w		12w		14w		17w		8w		12w		14w		17w	
Bio-transformation products	Conc (ng/g d.w.)	±SD	Conc (ng/g d.w.)	±SD	Conc (ng/g d.w.)	±SD	Conc (ng/g d.w.)	±SD	Conc (ng/g d.w.)	±SD	Conc (ng/g d.w.)	±SD	Conc (ng/g d.w.)	±SD	Conc (ng/g d.w.)	±SD
**Gills**																
PFBA	11.4	0.2474	15.8	0.34	1.63	0.04	2.71	0.06	15.1	0.33	18.8	0.41	3.22	0.07	4.52	0.10
PFPeA	0.02	0.0004	0.75	0.02	0.12	0.002	0.11	0.002	<LOQ	-	0.11	0.002	0.22	0.005	0.19	0.004
PFHxA	<LOQ	-	0.10	0.002	0.11	0.003	<LOQ	-	0.18	0.00	0.16	0.004	0.24	0.01	0.06	0.001
PFHpA	<LOQ	-	0.19	0.005	0.09	0.002	0.03	0.001	0.35	0.01	0.51	0.01	0.66	0.02	0.56	0.01
**Scaly**																
PFBA	<LOQ	-	<LOQ	-	3.45	0.08	1.31	0.03	<LOQ	-	0.63	0.01	51.6	1.13	16.1	0.35
PFPeA	0.03	0.001	0.08	0.002	0.19	0.004	0.07	0.002	0.03	0.001	0.09	0.002	0.22	0.005	0.03	0.001
PFHxA	<LOQ	-	0.01	0.0002	0.24	0.01	0.14	0.003	0.06	0.001	0.11	0.003	0.39	0.01	0.22	0.01
PFHpA	0.03	0.001	0.08	0.002	0.22	0.01	0.09	0.002	0.22	0.01	0.34	0.01	0.46	0.01	0.06	0.002
**Kidney**																
PFBA	<LOQ	-	97.5	2.08	19.5	0.42	13.3	0.28	<LOQ	-	204	4.35	104	2.22	68	1.45
PFPeA	0.32	0.007	0.61	0.01	1.35	0.03	2.86	0.06	0.32	0.01	0.51	0.01	0.9	0.02	2.52	0.06
PFHxA	<LOQ	-	<LOQ	-	1.55	0.04	<LOQ	-	0.99	0.02	1.12	0.03	1.31	0.03	1.42	0.03
PFHpA	0.11	0.003	0.39	0.01	0.69	0.02	<LOQ	-	1.82	0.04	1.96	0.05	2.32	0.06	2.4	0.06
**Muscle**																
PFBA	<LOQ	-	8.1	0.17	4.09	0.09	1.61	0.03	<LOQ	-	10.2	0.22	2.91	0.06	1.17	0.03
PFPeA	<LOQ	-	0.05	0.001	0.13	0.002	0.07	0.001	<LOQ	-	0.06	0.001	0.37	0.004	0.18	0.002
PFHxA	<LOQ	-	<LOQ	-	0.13	0.003	<LOQ	-	0.06	0.001	0.14	0.003	0.27	0.01	0.11	0.003
PFHpA	<LOQ	-	0.17	0.004	0.28	0.01	<LOQ	-	0.12	0.003	0.37	0.01	0.56	0.01	0.47	0.01
**Skin**																
PFBA	3.99	0.089	26	0.58	2.68	0.06	<LOQ	-	1.21	0.03	22.4	0.50	3.66	0.08	1.75	0.04
PFPeA	0.12	0.003	0.17	0.005	0.34	0.01	<LOQ	-	0.12	0.003	0.2	0.005	0.48	0.01	0.31	0.01
PFHxA	<LOQ	-	0.07	0.00	0.17	0.005	<LOQ	-	0.11	0.003	0.18	0.004	0.25	0.01	<LOQ	-
PFHpA	<LOQ	-	0.41	0.01	1.08	0.03	<LOQ	-	0.11	0.003	0.33	0.01	0.6	0.02	0.22	0.01
**Brain**																
PFBA	<LOQ	-	104	2.35	55	1.24	17.6	0.40	<LOQ	-	200	4.52	107	2.42	60.4	1.37
PFPeA	<LOQ	-	0.61	0.02	2.18	0.05	1.37	0.03	<LOQ	-	<LOQ	-	1.25	0.03	0.53	0.01

PFHxA	<LOQ	-	1.2	<LOQ	-	0.08	0.96	0.03	<LOQ	-	0.49	0.01	1.03	0.03	0.42	0.01
PFHpA	0.03	0.001	0.13	0.003	0.2	0.01	0.04	0.001	0.51	0.01	1.1	0.03	2.36	0.06	0.99	0.03
**Gonads**																
PFBA	<LOQ	-	42	0.97	39.6	0.91	22.1	0.51	10.9	0.25	62	1.43	57.2	1.32	32.1	0.74
PFPeA	<LOQ	-	0.27	0.01	0.62	0.01	0.43	0.01	<LOQ	-	<LOQ	-	0.96	0.02	0.31	0.01
PFHxA	<LOQ	-	<LOQ	-	2.77	0.07	3.22	0.08	0.09	0.002	3.17	0.08	5.34	0.14	0.11	0.003
PFHpA	0.07	0.002	0.21	0.01	0.39	0.01	0.07	0.001	0.3	0.01	0.42	0.01	0.79	0.02	0.42	0.01
**Liver**																
PFBA	1.00	0.022	58.5	1.28	3.87	0.08	2.99	0.07	<LOQ	-	80.6	1.76	20.4	0.44	10.3	0.22
PFPeA	<LOQ	-	0.05	0.001	0.28	0.01	0.47	0.01	<LOQ	-	0.02	0.0005	0.31	0.01	0.13	0.003
PFHxA	<LOQ	-	37.5	0.97	0.18	0.005	0.24	0.01	<LOQ	-	0.12	0.003	0.25	0.01	0.12	0.003
PFHpA	<LOQ	-	0.09	0.002	0.27	0.01	1.18	0.03	0.13	0.004	0.36	0.01	0.55	0.02	1.69	0.05
**Intestine**																
PFBA	<LOQ	-	9.00	0.21	5.15	0.12	1.69	0.04	<LOQ	-	7.6	0.18	2.25	0.05	1.53	0.04
PFPeA	<LOQ	-	0.15	0.004	0.18	0.004	0.14	0.003	0.12	0.003	0.12	0.003	0.23	0.01	0.21	0.01
PFHxA	<LOQ	-	0.10	0.003	0.22	0.01	<LOQ	-	<LOQ	-	0.09	0.003	0.16	0.01	0.16	0.01
PFHpA	<LOQ	-	0.07	0.002	0.18	0.01	<LOQ	-	0.09	0.003	0.19	0.01	0.36	0.01	0.28	0.01
**Gallbladder**																
PFBA	<LOQ	-	120	2.66	54	1.20	10.8	0.24	4.3	0.10	176	3.91	30	0.67	18.6	0.41
PFPeA	0.12	0.003	0.12	0.003	1.82	0.04	1.82	0.04	0.15	0.003	0.16	0.00	3.31	0.07	3.81	0.08
PFHxA	0.45	0.010	1.14	0.03	2.51	0.06	<LOQ	-	0.90	0.02	1.38	0.03	2.29	0.05	<LOQ	-
PFHpA	0.37	0.009	1.04	0.03	2.35	0.06	<LOQ	-	3.79	0.09	4.62	0.11	6.2	0.15	<LOQ	-

Note: <LOQ - result under quantification limit

foods MDPI

Table S10. Spearman correlation and p-values determined between PFOA and metabolites in gills

**Gills**		**Experiment 1**				**Experiment 2**			
		**PFPeA**	**PFHxA**	**PFHpA**	**PFOA**	**PFPeA**	**PFHxA**	**PFHpA**	**PFOA**
**PFBA**	Spearman Corr.	0.862	-0.316	0.200	0.600	-0.898	-0.400	-0.827	0.600
	p value	0.033	0.684	0.800	0.400	0.031	0.600	0.044	0.400
**PFPeA**	Spearman Corr.		0.738	1.000	-0.200		0.894	1.000	-0.927
	p value		0.262	0.000	0.800		0.043	0.000	0.020
**PFHxA**	Spearman Corr.			0.738	-0.105			0.200	-0.800
	p value			0.042	0.895			0.800	0.200
**PFHpA**	Spearman Corr.				-0.873				-0.877
	p value				0.016				0.037

Strong Spearman correlation values

Table S11. Spearman correlation and p-values determined between PFOA and metabolites in Kidney

Kidney		Experiment 1				Experiment 2			
		PFPeA	PFHxA	PFHpA	PFOA	PFPeA	PFHxA	PFHpA	PFOA
PFBA	Spearman Corr.	0.813	0.258	0.600	-0.200	0.846	0.200	0.200	-0.200
	p value	0.047	0.742	0.400	0.800	0.039	0.800	0.800	0.800
PFPeA	Spearman Corr.		0.747	-0.200	-1.000		1.000	1.000	-1.000
	p value		0.050	0.800	0.000		0.000	0.000	0.000
PFHxA	Spearman Corr.			0.775	-0.258			1.000	-1.000
	p value			0.025	0.742			0.000	0.000
PFHpA	Spearman Corr.				0.891				-1.000
	p value				0.033				0.000

Strong Spearman correlation values

Table S12. Spearman correlation and p-values determined between PFOA and metabolites in brain

Brain		Experiment 1				Experiment 2			
		PFPeA	PFHxA	PFHpA	PFOA	PFPeA	PFHxA	PFHpA	PFOA
PFBA	Spearman Corr.	0.400	0.800	0.800	0.400	0.895	0.800	0.800	-0.200
	p value	0.600	0.200	0.200	0.600	0.025	0.200	0.200	0.800
PFPeA	Spearman Corr.		0.899	0.600	-0.600		0.932	0.832	-0.738
	p value		0.023	0.323	0.400		0.033	0.036	0.262
PFHxA	Spearman Corr.			1.000	0.000			1.000	-0.400
	p value			0.000	1.000			0.000	0.600
PFHpA	Spearman Corr.				1.000				-0.869
	p value				0.002				0.027

Strong Spearman correlation values

Table S13. Spearman correlation and p-values determined between PFOA and metabolites in gonads

Gonads		Experiment 1				Experiment 2			
		PFPeA	PFHxA	PFHpA	PFOA	PFPeA	PFHxA	PFHpA	PFOA
PFBA	Spearman Corr.	0.822	-0.105	0.600	0.400	0.895	0.500	0.632	-0.200
	p value	0.041	0.895	0.400	0.600	0.011	0.222	0.368	0.800
PFPeA	Spearman Corr.		0.792	0.882	-0.600		0.832	0.833	-0.738
	p value		0.032	0.033	0.400		0.036	0.037	0.262
PFHxA	Spearman Corr.			-0.316	-0.949			0.949	-0.833
	p value			0.684	0.041			0.021	0.038
PFHpA	Spearman Corr.				-0.858				-0.872
	p value				0.042				0.036

Strong Spearman correlation values

Table S14. Spearman correlation and p-values determined between PFOA and metabolites in liver

Liver		Experiment 1				Experiment 2			
		PFPeA	PFHxA	PFHpA	PFOA	PFPeA	PFHxA	PFHpA	PFOA
PFBA	Spearman Corr.	0.822	0.800	0.200	-0.400	0.807	0.800	0.200	-0.200
	p value	0.043	0.200	0.800	0.600	0.049	0.200	0.800	0.800
PFPeA	Spearman Corr.		0.926	1.000	-0.869		0.883	1.000	-0.889
	p value		0.041	0.000	0.045		0.048	0.000	0.032
PFHxA	Spearman Corr.			0.400	-0.200			0.400	-0.400
	p value			0.600	0.800			0.600	0.600
PFHpA	Spearman Corr.				-0.823				-1.000
	p value				0.020				0.000

Strong Spearman correlation values

Table S15. Spearman correlation and p-values determined between PFOA and metabolites in gallbladder

Gallbladder		Experiment 1				Experiment 2			
		PFPeA	PFHxA	PFHpA	PFOA	PFPeA	PFHxA	PFHpA	PFOA
PFBA	Spearman Corr.	0.783	0.600	0.600	-0.200	0.897	0.600	0.600	-0.200
	p value	0.039	0.400	0.400	0.800	0.020	0.400	0.400	0.800
PFPeA	Spearman Corr.		-0.792	-0.200	-1.000		-0.804	-0.200	-1.000
	p value		0.048	0.800	0.000		0.033	0.800	0.000
PFHxA	Spearman Corr.			1.000	0.200			1.000	0.200
	p value			0.000	0.800			0.000	0.800
PFHpA	Spearman Corr.				0.855				0.861
	p value				0.044				0.048

Strong Spearman correlation values

Table S16. Spearman correlation and p-values determined between PFOA and metabolites in intestine

Intestine		Experiment 1				Experiment 2			
		PFPeA	PFHxA	PFHpA	PFOA	PFPeA	PFHxA	PFHpA	PFOA
PFBA	Spearman Corr.	0.800	0.738	0.738	0.400	0.400	0.316	0.400	0.400
	p value	0.200	0.262	0.262	0.600	0.600	0.684	0.600	0.600
PFPeA	Spearman Corr.		0.949	0.949	1.000		0.949	1.000	-0.930
	p value		0.020	0.020	0.000		0.031	0.000	0.024
PFHxA	Spearman Corr.			1.000	0.211			0.949	-0.738
	p value			0.000	0.789			0.033	0.262
PFHpA	Spearman Corr.				0.789				-0.847
	p value				0.011				0.029

Strong Spearman correlation values

Table S17. Spearman correlation and p-values determined between PFOA and metabolites in muscle

Muscle		Experiment 1				Experiment 2			
		PFPeA	PFHxA	PFHpA	PFOA	PFPeA	PFHxA	PFHpA	PFOA
PFBA	Spearman Corr.	0.400	0.632	0.738	0.800	0.400	0.800	0.400	0.400
	p value	0.600	0.368	0.232	0.200	0.600	0.200	0.600	0.600
PFPeA	Spearman Corr.		0.949	0.632	0.000		0.866	1.000	-0.600
	p value		0.041	0.368	1.000		0.037	0.000	0.400
PFHxA	Spearman Corr.			0.833	0.316			0.794	0.000
	p value			0.017	0.684			0.037	1.000
PFHpA	Spearman Corr.				0.738				-0.795
	p value				0.022				0.045

Strong Spearman correlation values

Table S18. Spearman correlation and p-values determined between PFOA and metabolites in skin

Skin		Experiment 1				Experiment 2			
		PFPeA	PFHxA	PFHpA	PFOA	PFPeA	PFHxA	PFHpA	PFOA
PFBA	Spearman Corr.	0.400	0.211	0.211	0.400	0.400	0.600	0.800	-0.200
	p value	0.600	0.789	0.789	0.600	0.600	0.400	0.200	0.800
PFPeA	Spearman Corr.		0.949	0.949	1.000		0.793	0.807	-0.800
	p value		0.021	0.025	0.000		0.042	0.048	0.200
PFHxA	Spearman Corr.			1.000	0.949			0.839	0.200
	p value			0.000	0.020			0.040	0.800
PFHpA	Spearman Corr.				0.949				-0.855
	p value				0.017				0.046

Strong Spearman correlation values

Table S19. Spearman correlation and p-values determined between PFOA and metabolites in scaly

Skaly		Experiment 1				Experiment 2			
		PFPeA	PFHxA	PFHpA	PFOA	PFPeA	PFHxA	PFHpA	PFOA
PFBA	Spearman Corr.	0.832	0.949	0.949	0.316	0.932	1.000	0.847	-0.800
	p value	0.036	0.051	0.051	0.684	0.027	0.000	0.039	0.020
PFPeA	Spearman Corr.		0.800	0.897	0.800		0.632	0.949	-0.105
	p value		0.200	0.038	0.200		0.368	0.051	0.895
PFHxA	Spearman Corr.			1.000	0.821			0.899	-0.825
	p value			0.000	0.040			0.0.22	0.044
PFHpA	Spearman Corr.				0.833				0.867
	p value				0.045				0.032

Strong Spearman correlation values

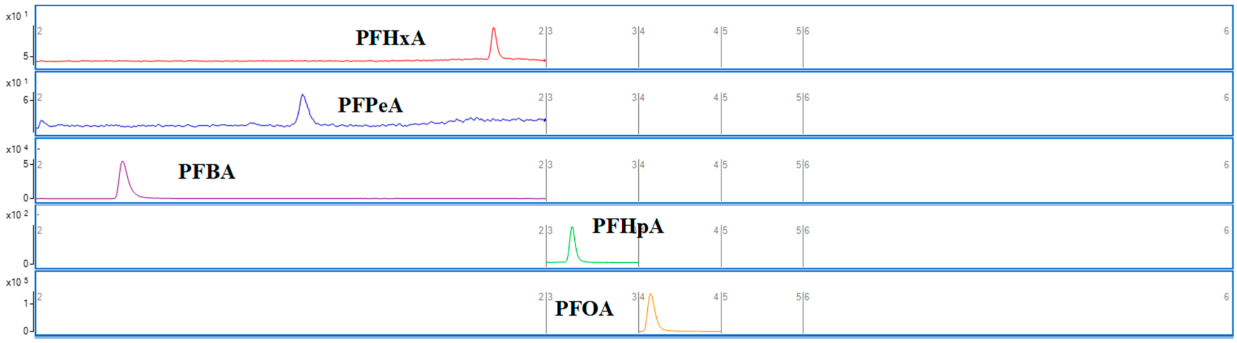

Figure S1. MRM chromatogram obtained for PFOA and selected metabolites (10 µg/L).

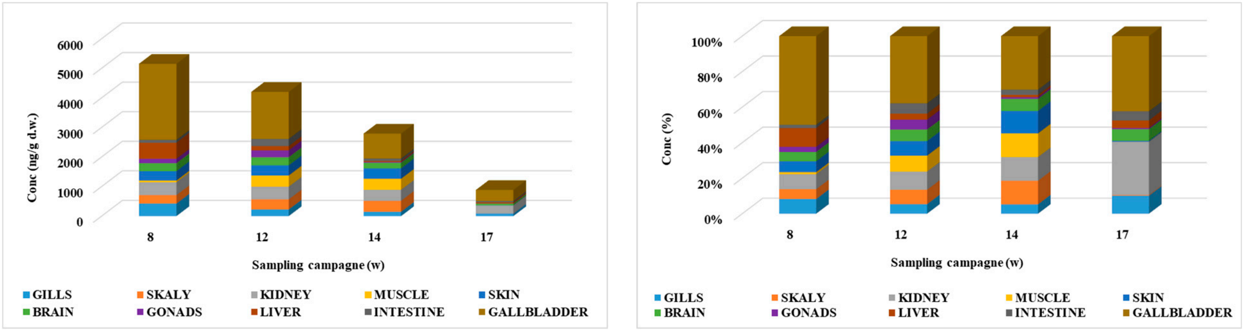

Figure S2. The total concentration of PFOA (a) and the percentage distribution in organs (b) in Experiment 1.

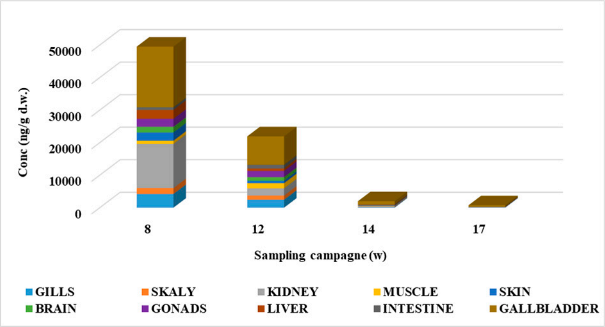

(**a**)

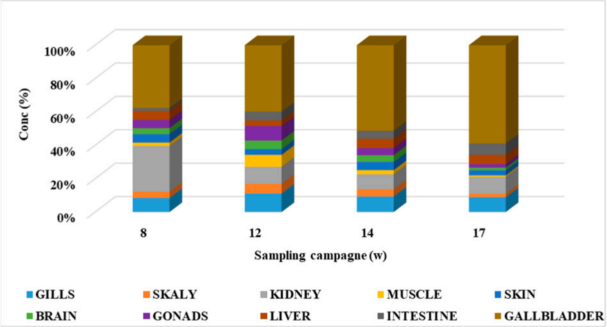

(**b**)

Figure S3. The total concentration of PFOA (a) and the percentage distribution in organs (b) in Experiment 2.

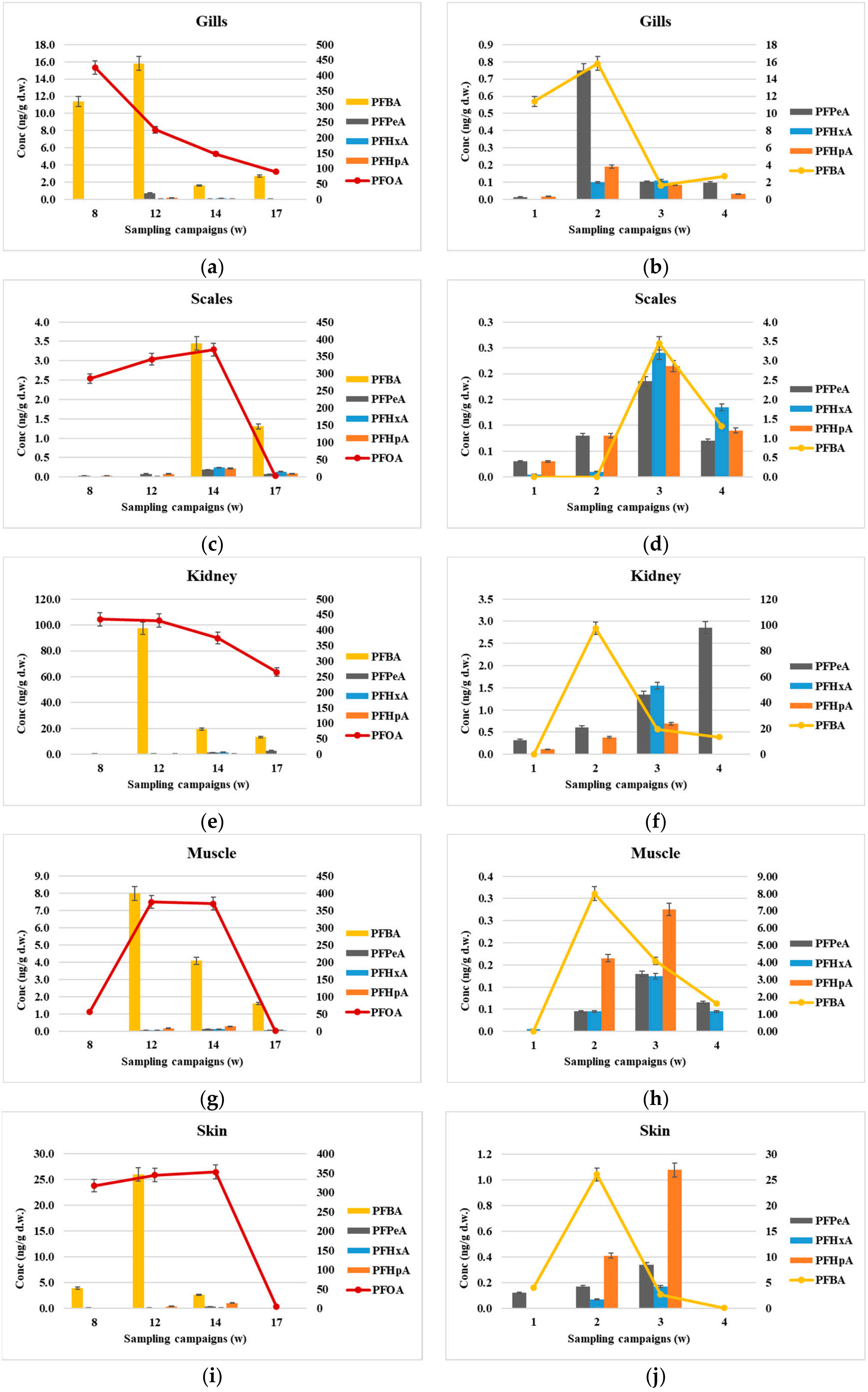

Gills
Conc (ng/g d.w.)
Sampling campaigns (w)
PFBA
PFPeA
PFHxA
PFHpA
PFOA
(a)
Gills
(b)
Scales
(c)
Scales
(d)
Kidney
(e)
Kidney
(f)
Muscle
(g)
Muscle
(h)
Skin
(i)
Skin
(j)

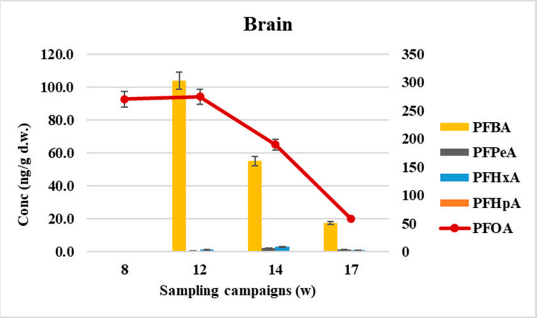

(k)

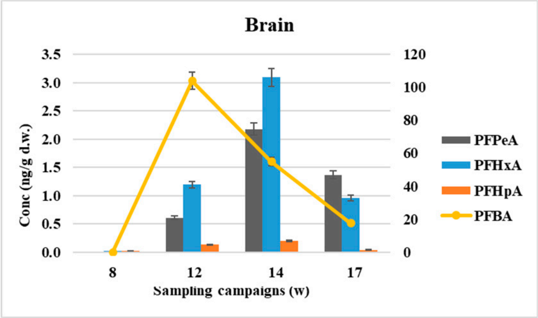

(l)

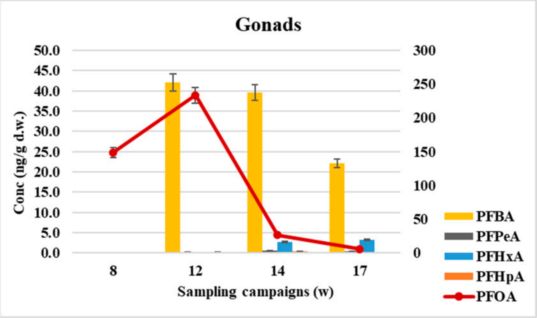

(m)

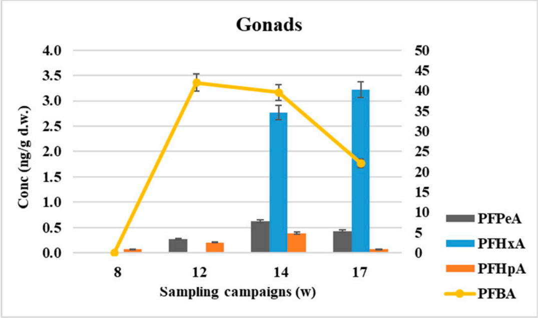

(n)

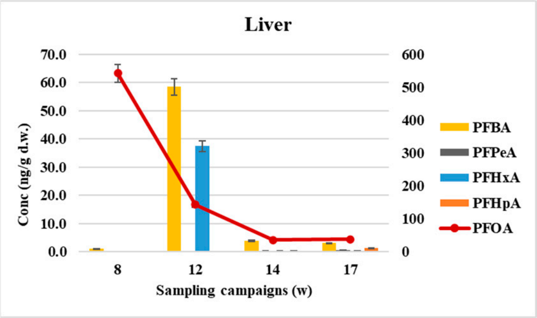

(o)

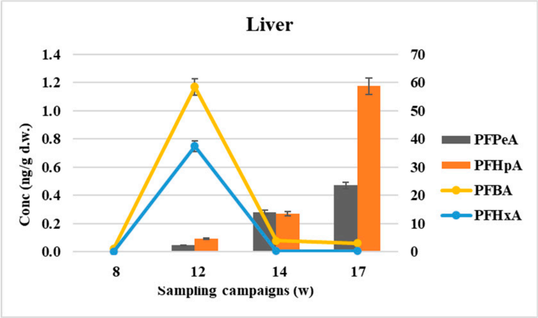

(p)

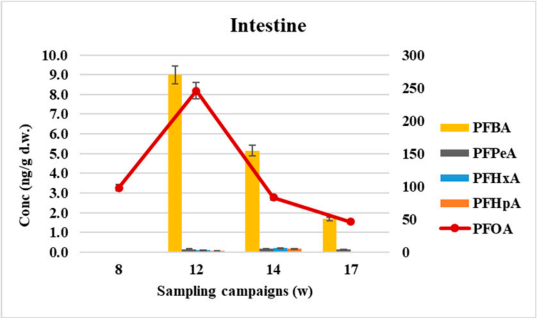

(q)

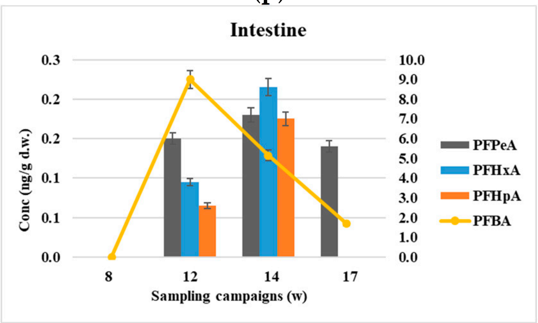

(r)

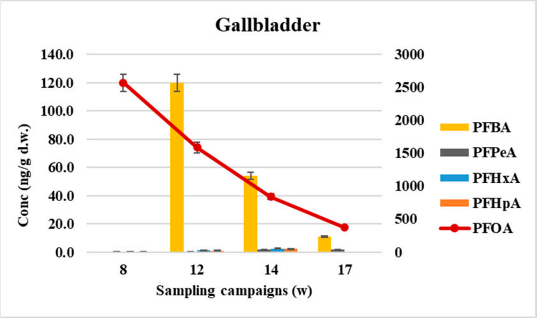

(s)

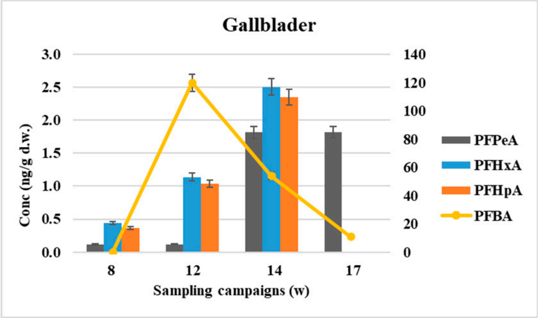

(t)

Figure S4. PFOA variation and biotransformation products evolution in fish organs in Experiment 1: gills (a), (b); scales (c), (d); kidney (e), (f); muscle (g), (h); skin (i), (j); brain (k), (l); gonads (m), (n); liver (o), (p); intestine (q), (r); gallbladder (s), (t).

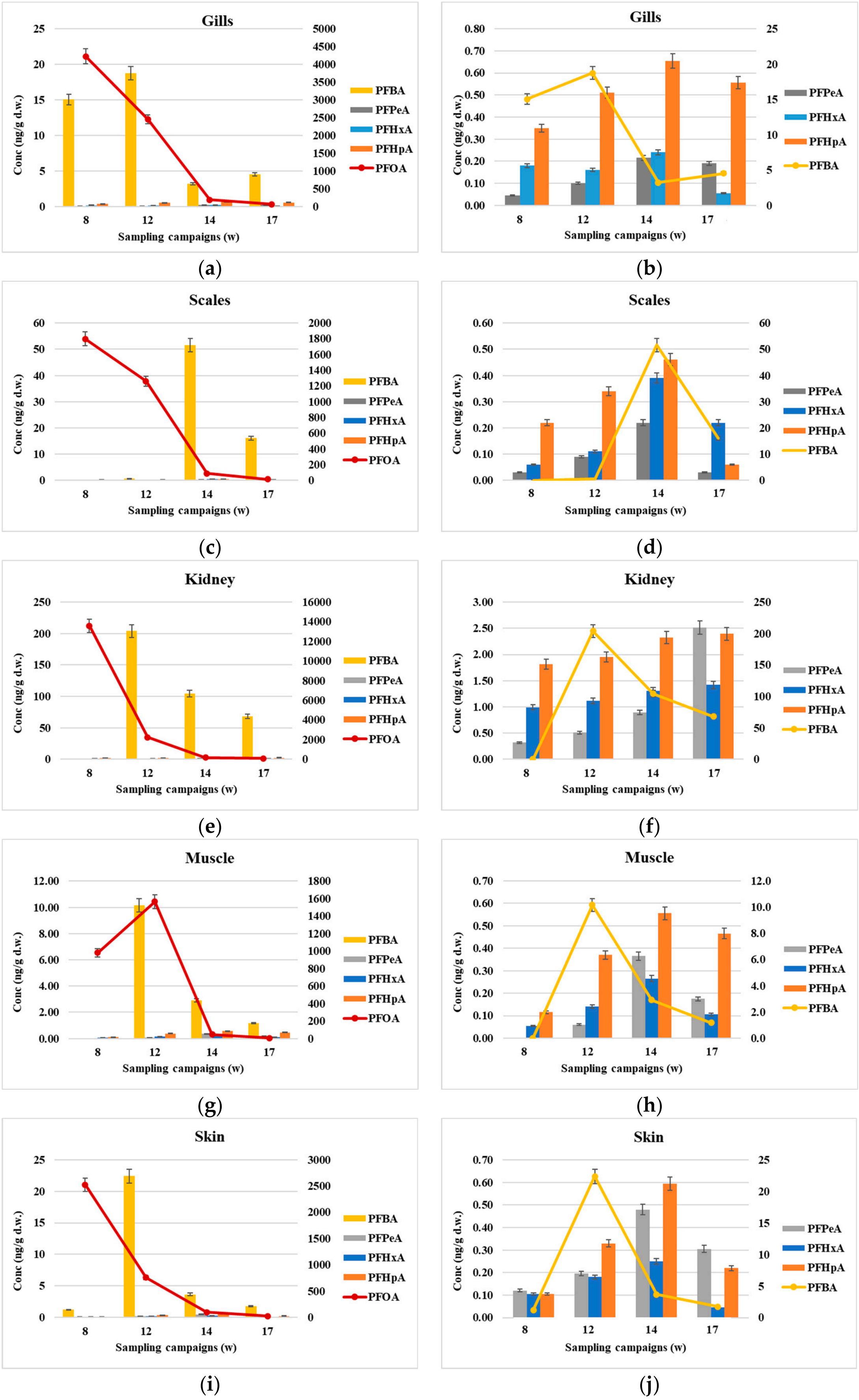
Gills
Conc (ng/g d.w.)
Sampling campaigns (w)
PFBA
PFPeA
PFHxA
PFHpA
PFOA
(a)
Gills
PFPeA
PFHxA
PFHpA
PFBA
(b)
Scales
PFBA
PFPeA
PFHxA
PFHpA
PFOA
(c)
Scales
PFPeA
PFHxA
PFHpA
PFBA
(d)
Kidney
PFBA
PFPeA
PFHxA
PFHpA
PFOA
(e)
Kidney
PFPeA
PFHxA
PFHpA
PFBA
(f)
Muscle
PFBA
PFPeA
PFHxA
PFHpA
PFOA
(g)
Muscle
PFPeA
PFHxA
PFHpA
PFBA
(h)
Skin
PFBA
PFPeA
PFHxA
PFHpA
PFOA
(i)
Skin
PFPeA
PFHxA
PFHpA
PFBA
(j)

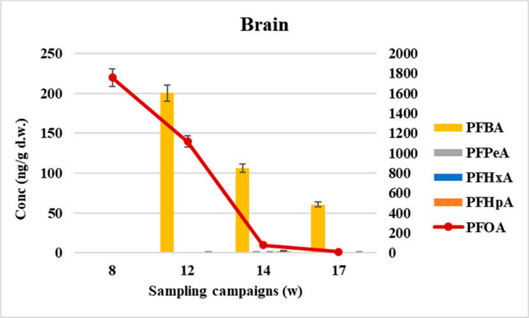

(k)

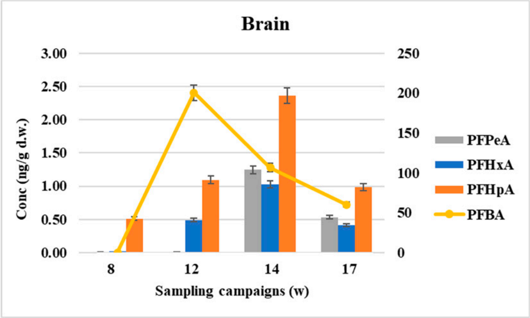

(l)

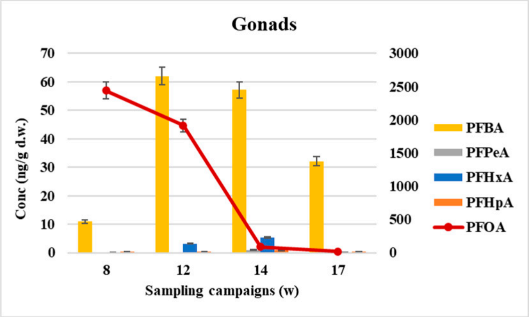

(m)

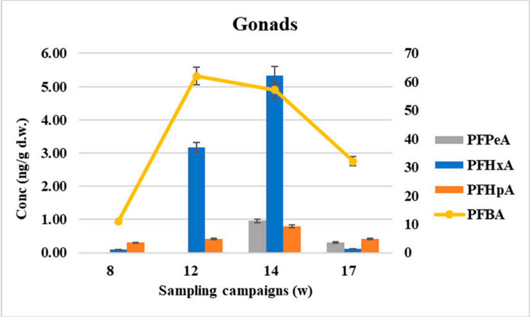

(n)

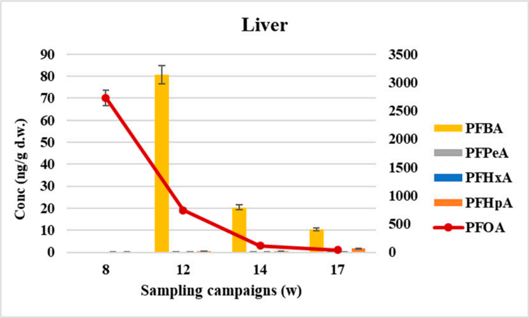

(o)

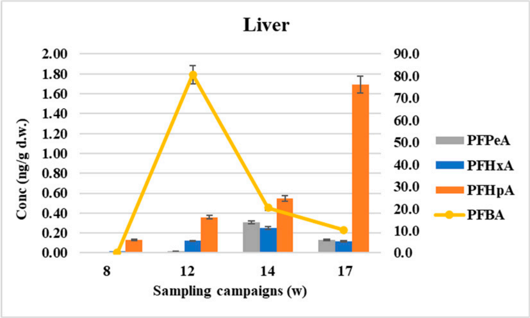

(p)

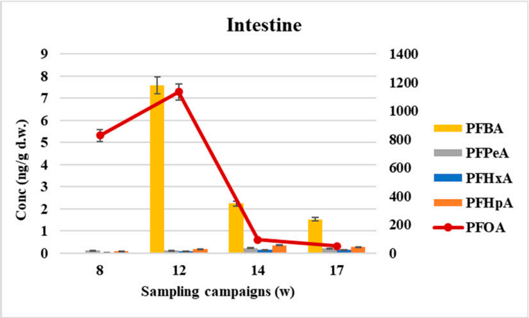

(q)

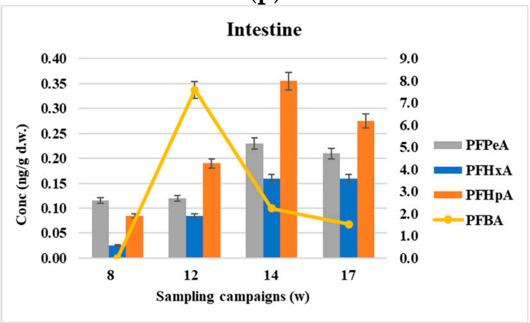

(r)

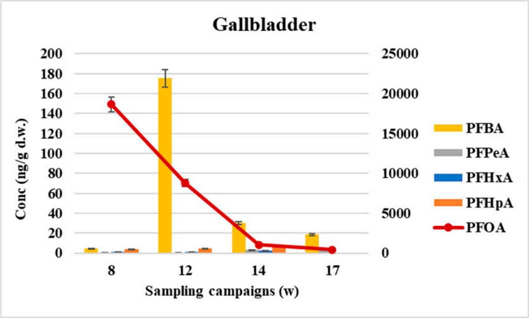

(s)

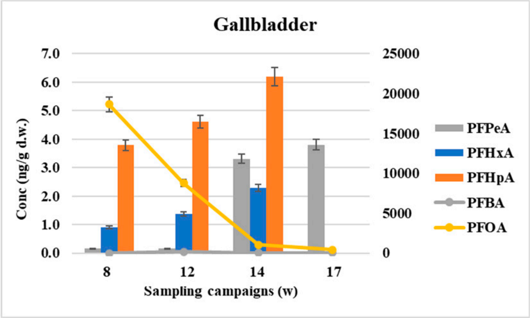

(t)

Figure S5. PFOA variation and biotransformation products evolution in fish organs in Experiment 2: gills (a), (b); scales (c), (d); kidney (e), (f); muscle (g), (h); skin (i), (j); brain (k), (l); gonads (m), (n); liver (o), (p); intestine (q), (r); gallbladder (s), (t).

References

60. Zhang, L.; Niu, J.; Wang, Y.; Shi, J; Huang, Q. Chronic Effects of PFOA and PFOS on Sexual Reproduction of Freshwater Rotifer *Brachionus calyciflorus*. Chemosphere. **2014**, *114*, 114–120.
61. Zhai, Y.; Xia, X.; Zhao, X.; Dong, H.; Zhu, B.; Xia, N.; Dong, J. Role of Ingestion Route in the Perfluoroalkyl Substance Bioaccumulation by *Chironomus plumosus* Larvae in Sediments Amended with Carbonaceous Materials. J. Hazard. Mater. **2016**, *302*, 404–414.
62. Marziali, L.; Rosignoli, F.; Valsecchi, S.; Polesello, S.; Stefani, F. Effects of Perfluoralkyl Substances on a Multigenerational Scale: A Case Study with *Chironomus riparius* (Diptera, Chironomidae). Environ Toxicol. Chem. 2019, 38(5), 988-999.
63. Ding, G.H.; Frömel, T.; van den Brandhof, E.J.; Baerselman, R.; Peijnenburg, W.J.G.M. Acute toxicity of poly- and perfluorinated compounds to two cladocerans, *Daphnia magna* and *Chydorus sphaericus*. Environ. Toxicol. Chem. **2012**, *31*, 605–610.
64. Yang, S.; Xu, F.; Wu, F.; Wang, S.; Zheng, B. Development of PFOS and PFOA criteria for the protection of freshwater aquatic life in China. Sci. Total Environ. **2014**, *470–471*, 677–683.
65. Sanderson, H.; Boudreau, T.M.; Mabury, S.A.; Solomon, K.R. Impact of Perfluorooctanoic Acid on the Structure of the Zooplankton Community in Indoor Microcosms. Aquat. Toxicol. 2003, 62(3), 227-234.
66. Barmentlo, S.H.; Stel, J.M.; van Doorn, M.; Eschauzier, C.; de Voogt, P.; Kraak, M.H. Acute and chronic toxicity of short chained perfluoroalkyl substances to *Daphnia magna*. Environ. Pollut. **2015**, *198*, 47–53.
67. Boudreau, T.M. Toxicity of Perfluorinated Organic Acids to Selected Freshwater Organisms Under Laboratory and Field Conditions, M.S. Thesis, University of Guelph, Ontario, Canada. 2002, 145.
68. Zhang, J.; Wang, B.; Zhao, B.; Li, Y.; Zhao, X.; Yuan, Z. Blueberry Anthocyanin Alleviate Perfluorooctanoic Acid-Induced Toxicity in Planarian (*Dugesia japonica*) by Regulating Oxidative Stress Biomarkers, ATP Contents, DNA Methylation and mRNA Expression. Environ. Pollut. **2019**, *245*, 957–964.
69. Hazelton, P.D.; Cope, W.G.; Pandolfo, T.J.; Mosher, S.; Strynar, M.J.; Barnhart, M.C.; Bringolf, R.B. Partial life-cycle and acute toxicity of perfluoroalkyl acids to freshwater mussels. Environ. Toxicol. Chem. **2012**, *31*, 1611–1620.
70. Ji, K.; Kim, Y.; Oh, S.; Ahn, B.; Jo, H.; Choi, K. Toxicity of perfluorooctane sulfonic acid and perfluorooctanoic acid on freshwater macroinvertebrates (*Daphnia magna* and *Moina macrocopa*) and fish (*Oryzias latipes*). Environ. Toxicol. Chem. **2008**, *27*, 2159–2168.
71. Li, M.H. Toxicity of perfluorooctane sulfonate and perfluorooctanoic acid to plants and aquatic invertebrates. Environ. Toxicol. **2009**, *24*, 95–101.
72. Manera, M.; Giari, L.; Vincenzi, F.; Guerranti, C.; DePasquale, J.A.; Castaldelli, G. Texture Analysis in Liver of Common Carp (*Cyprinus carpio*) Sub-Chronically Exposed to Perfluorooctanoic Acid. Ecol. Indic. **2017**, *81*, 54–64.
73. Oakes, K.D.; Sibley, P.K.; Solomon, K.R.; Mabury, S.A.; Van der Kraak, G.J. Impact of perfluorooctanoic acid on fathead minnow (*Pimephales promelas*) fatty acyl-CoA oxidase activity, circulating steroids, and reproduction in outdoor microcosms. Environ. Toxicol. Chem. **2004**, *23*, 1912–1919.
74. Ye, L.; Wu, L.L.; Jiang, Y.X.; Zhang, C.J.; Chen, L. Toxicological study of PFOS/PFOA to zebrafish (*Danio rerio*) embryos. Huan Jing Ke Xue **2009**, *30*, 1727–1732.
